# Cancer Survivors Living in Rural Settings: A Qualitative Exploration of Concerns, Positive Experiences and Suggestions for Improvements in Survivorship Care

**DOI:** 10.3390/curroncol30080533

**Published:** 2023-08-02

**Authors:** Irene Nicoll, Gina Lockwood, Margaret I Fitch

**Affiliations:** 1 Health Care Consultant, Toronto, ON, Canada; nicollirene4@gmail.com; 2 Biostatistician Consultant, Toronto, ON, Canada; lockwo12@gmail.com; 3 Bloomberg Faculty of Nursing, University of Toronto, Toronto, ON M4C 4V9, Canada

**Keywords:** cancer, rural-dwelling, qualitative analysis, cancer survivor perspectives

## Abstract

In Canada, the number of cancer survivors continues to increase. It is important to understand what continues to present difficulties after the completion of treatment from their perspectives. Various factors may present barriers to accessing help for the challenges they experience following treatment. Living rurally may be one such factor. This study was undertaken to explore the major challenges, positive experiences and suggestions for improvement in survivorship care from rural-dwelling Canadian cancer survivors one to three years following treatment. A qualitative descriptive analysis was conducted on written responses to open-ended questions from a national cross-sectional survey. A total of 4646 individuals living in rural areas responded to the survey. Fifty percent (2327) were male, and 2296 (49.4%) were female; 69 respondents were 18 to 29 years (1.5%); 1638 (35.3%) were 30 to 64 years; and 2926 (63.0%) were 65 years or older. The most frequently identified major challenges (n = 5448) were reduced physical capacity and the effects of treatment. Positive experiences included family and friend support and positive self-care practices. The suggestions for improvements focused on the need for better communication and information about self-care, side effect management, and programs and services, with more programs available locally for practical and emotional support.

## 1. Introduction

By the year 2025, it is anticipated that there will be more than 25 million cancer survivors worldwide [1,2]. Cancer diagnosis and treatment can leave individuals with ongoing physical, emotional and practical challenges, which can have a profound impact on their quality of life and well-being [3]. Understanding the experiences of survivors and the evidence to guide practice during survivorship has grown considerably over the past decade [4,5]. But, given the heterogeneous nature of this population, providing tailored survivorship care is a multifaceted, complex process [6]. As the number of cancer survivors continues to grow, it is important to understand, from their perspective, what continues to present difficulties for them after the completion of treatment and whether they are able to access assistance for those difficulties.

One of the potential barriers to accessing care for cancer patients has been identified as living in a rural setting. Rural-dwelling cancer patients have been reported as having higher mortality [6,7] and poorer overall outcomes compared to their urban-dwelling counterparts [8,9]. Living in a rural setting has been reported as a barrier to screening for cancer [10], early diagnosis and treatment [11,12,13], mental health care [14], rehabilitation and psychosocial services [15], and palliative care [16]. Specifically, rural-dwelling cancer patients have reported an increased burden from travel and the accompanying travel costs, as well as a lack of relevant cancer-related and service information [17,18]. These previous studies have focused on individuals prior to diagnosis and during cancer treatment. However, perspectives from survivors about access to care following the completion of cancer treatment have received relatively little attention [5,19,20]. With the growing number of survivors, there is an opportunity to learn more about their perspectives on survivorship, its challenges and barriers to care.

Less than one-fifth of Canadians live in rural/remote settings, and approximately seven million are in regions with <1000 individuals and a population density of <400 per square kilometer [21]. Both positive and negative aspects have been described by residents about living in rural settings. Positive aspects include physical beauty, peaceful living and privacy [6,22], while negative aspects include issues with travel and financial costs for healthcare, lack of personal privacy in small communities and feelings of isolation [15]. Additionally, individuals living rurally can hold a strong attitude of stoicism, orientation to a self-sufficient lifestyle and be less likely to ask for help [23,24].

Survivorship is a new and growing consideration for Canadian cancer control, and it is important to understand what might constitute unique aspects of survivors’ needs. Little work has been completed to understand the perspectives of cancer survivors living in a rural setting. Understanding the perspectives of cancer survivors who live in rural locations regarding their survivorship care and access to relevant follow-up is important for the purpose of organizing appropriate service delivery. The Canadian Transitions study established a publicly available dataset and the opportunity to conduct secondary analyses regarding the experiences of cancer survivors. The secondary analysis reported in this paper is a qualitative analysis of the open-ended question responses from respondents in the Canadian Transitions Study who reported living in a rural location.

## 2. Methods

The need for Canadian data regarding survivors’ perspectives to inform cancer system developments in survivorship care led to the Canadian Partnership Against Cancer’s Transitions Survey [25]. This pan-Canadian survey was distributed across ten provinces to a randomly selected cancer registry sample of 40,790 cancer survivors between one and three years after the completion of cancer treatment. Each of the ten provincial cancer registries selected a random sample of cancer survivors based on their population, one to three years post-treatment. The survey was designed for cancer survivors most apt to be followed in the community, based on their cancer type, to identify their needs and explore their experiences with follow-up care. The period of one to three years allowed survivors to have time to experience follow-up care. Eligibility included adult survivors over the age of 35 with breast, prostate, colorectal and melanoma diseases with no metastatic spread and selected hematological cancers (e.g., Hodgkin’s Lymphoma, diffuse B cell lymphoma, acute myelogenous leukemia, acute lymphocytic leukemia), and those aged 18 to 34 years, with all non-metastatic cancer types except testes, where the metastatic disease was included because of its high survival rate. The survey was lengthy and contained many aspects of survivorship care using both closed- and open-ended items. The intention of the original work was to create a database that would be publicly available and accessible for secondary analyses. The detailed report of the original national survey was published previously [25].

This paper highlights a secondary analysis of open-ended question responses from rural-dwelling respondents about the biggest challenge they faced since completing cancer treatment, their positive experiences during care and their suggestions for improvements. The first question was intended to learn what survivors found most troublesome or difficult during the early survivorship period. The second question was intended to learn what they valued or appreciated about survivorship care, and the third focused on what was missing from their care that would be beneficial to others if it were available. A rural setting was defined as living in villages or towns with 10,000 or fewer residents or on an acreage, ranch or farm.

Ethics approval was granted by respective ethics boards from the ten provincial cancer agencies participating in survey distribution (Alberta = HREBA.CC-16-0025; Manitoba = HS19571(H2016.114); Nova Scotia = NSHA REB ROMEO #1021104; Newfoundland = #2016.080; Saskatchewan = BEH#16–79; Ontario/Hamilton#1528; no assigned numbers for New Brunswick, Prince Edward Island, Quebec or British Columbia reviews). Participants provided informed consent prior to completing the survey.

### Qualitative Analysis

The analysis used a descriptive qualitative approach conducting a conventional content analysis [26,27] as an exploration. The intention was to determine the types and frequencies of topics respondents mentioned. Verbatim written responses from the open-ended questions were entered into an Excel spreadsheet, and analysis was conducted for each question separately. No preconceived notions about the content categories were imposed. The two team members who engaged in the analysis have extensive qualitative training and experience and worked in oncology settings. They designed the coding framework after reading the written comments independently and identifying content topics within the responses. The objective was to stay as close to the respondents wording as possible. The researchers discussed their observations and arrived at a consensus about the coding categories. This set of categories was then used to code the data. The third author reviewed the analysis and resolved any outstanding disagreements.

All responses were coded by the two team members, and any disagreements were discussed to achieve consensus. Content in each category was reviewed by both team members, and categories were grouped into broader conceptual domains. Coded responses were grouped by age to explore if there were different perspectives being shared. For this analysis, adolescents and young adults (AYA) were defined as between 18 and 29 years, adults were defined as between 30 and 64 years, and older adults were defined as 65 years and older. Given that each age group is facing different life tasks, there may be different perspectives about living in a rural setting that could influence survivorship challenges. The results for those respondents who did not disclose their ages were not included in this analysis.

## 3. Results

### 3.1. Demographic Characteristics 

A total of 4646 individuals from rural areas responded to the survey. Fifty percent (2327) were male, 2296 (49.4%) were female, and 23 (0.5%) preferred not to answer (Table 1). A total of 69 respondents were 18 to 29 years (1.5%); 1638 (35.3%) were 30 to 64 years; and 2926 (63.0%) were 65 years or older. Thirteen respondents preferred not to disclose their ages, and their comments were not included in the analysis. Close to 75.0% of respondents had breast, prostate or colorectal cancers. Less than 10% reported living with metastases, and over 30.0% reported comorbidities. A total of 1819 (39.2%) resided in areas between 2000 and 10,000 residents; 1452 (31.3%) lived on an acreage, ranch, or farm; and 1375 (29.6%) lived in areas with 2000 or fewer residents. Approximately 40.0% of the responses to all questions were submitted by those living in areas between 2000 and 10,000 residents, approximately 30.0% were submitted by those living on acreages, and 30.0% were by those living in areas with 2000 or fewer residents.

### 3.2. Perspectives in Written Comments

The written comments from respondents ranged in length and depth. Many simply wrote a word (e.g., “*pain*”, “*fatigue*”) while others wrote a phrase (“*the pain was unbearable after radiation*”*,* “*constant fatigue and really low energy*“) or several sentences. Respondents often described several topics within their written comments to one question. The reporting below focuses on the responses within each of the three open-ended questions separately.

### 3.3. Major Challenges

There were 5448 major challenges identified in the written comments. Two-thirds (64.1%) focused on physical concerns that included physical capacity (pain, numbness, swelling), other effects of therapy (long recovery, comorbidities, infection) and therapy effects (due to medication, chemotherapy, radiation) (Table 2). Physical capacity encompassed concerns related to fatigue/exhaustion, lack of stamina/energy, weakness/regaining strength and restricted mobility. Respondents’ comments illustrated the difficulties faced: “*there was lots of stomach upsets, pain, swelling in my right arm, unable to sleep.*”; “*unable to do heavy farm work after surgery.*”; “*numbness of lower limbs and superior*; *loss of motor skills in upper limbs*”; and “*loss of energy, difficulty with endurance fatigue*”. Physical capacity and pain were experienced with changes or restrictions in body function. “*Abdominal pain; change in bowel habits, occasional incomplete small bowel obstruction*”, wrote one respondent. “*I was burned badly by the radiation, which was extremely painful and took a long time to heal*”*,* said another.

Smaller proportions of respondents identified emotions as major challenges. Emotional concerns were reported in 13.1% (713) of the identified major challenges. These concerns included difficulties with coping, depression and anxiety, fear of recurrence, and fear of death. The difficulty of coping with uncertainty emerged as a common theme. “*Coming to grips with the realization that no definite signs that cancer was in full remission and could reoccur at any time*”, wrote one respondent. Close to seven percent (6.9%, 376) of the comments regarding major challenges were related to service delivery, including lack of clear information, issues with communication and coordination among healthcare providers, disappointment with dismissive or inattentive healthcare providers, prolonged wait times, and lack of access to care and services. “*Absolutely no communication in regards to ease of obtaining a follow-up colonoscopy*”, wrote one respondent. “*My family doctor said my follow-up care was up to surgeon—surgeon said it was up to family doctor*”, wrote another. “*I found that I was cut loose with almost no support, especially from the oncology department*”. Additionally, lifestyle adjustments were the focus of 344 (6.4%) major challenge comments. These included adjustments the respondents found they were required to make to their daily habits, such as difficulties or restrictions with driving and dressing, as well as adjustments in diet, sleep and personal habits (i.e., using sunscreen). Lifestyle comments submitted by older adults 65 years or older were 61.9% (213) of the total.

### 3.4. Positive Experiences

Rural respondents wrote comments about 3561 positive experiences (Table 3). Receiving care by excellent, supportive and attentive healthcare providers was the topic of approximately a quarter (22.7%, 807) of these comments. “*Knowing the support of my doctor and the doctor and nurses at the cancer centre was a phone call away was the most positive experience I could wish for*”, wrote one respondent. Over a fifth of the comments (21.3%, 759) highlighted advice to other survivors and self-care strategies they practiced. The self-care strategies included the importance of maintaining a positive attitude, staying healthy, and listening to and trusting the medical team. One respondent wrote: “*stay as positive as you are able as I think the mental state of a person is certainly part of the battle*”. “*Take things one day at a time. Don’t try to over analyze things. Listen to your doctor*”, which was a common theme. Self-care advice included, “*check your skin regularly for spots*” and “*getting started with an exercise program sure helps with strength and helping to feel confident*”.

Comments on the value of support from family, friends, other cancer survivors and community/support groups accounted for 20.7% (736) of the total positive experience comments. “*Having someone to talk to and knowing they understand what you are going through*”, wrote a respondent. “*I had a very supportive wife and church group that helped me through the hard times*”, wrote another. Other positive experiences included access to and the existence of timely follow-up care; clear, timely and easy communication with healthcare providers; and appreciation for successful treatment and good cancer treatment experiences overall. These survivors valued the timely access they had to diagnosis, treatment and follow-up care, as well as being cared for by healthcare professionals who were knowledgeable and compassionate. “*My nurse practitioner explained everything and continues to counsel me*”*,* wrote one respondent. Some respondents (130, 3.7%) wrote about the positive effects of restorative therapies, alternative therapies (e.g., naturopathy, massage, meditation, acupuncture) and receiving guidance about diet, exercise and sleep. Although less than 5.0% of all respondents, some indicated they experienced nothing positive during their survivorship care.

### 3.5. Suggestions for Improvement

The majority of the 3181 suggestions for improvement focused on topics related to information and communication, support and self-care, and follow-up care (Table 4). Respondents wrote about the need for better information on self-care, cancer prevention, care plans, management of side effects and support programs and services (25.1%, 798). “*Tell me what is (normal) acceptable pain to experience after I get home from surgery*”, wrote one respondent. “*I suffered too much, too long, then found out there were problems*.” Over two-thirds of the support and self-care comments (18.0%, 574) highlighted the need for improvements in family, peer and group support and practical support, including financial aid, return to work assistance, and help with chores and travel. “*Cannot emphasize enough that the support of family and particularly spouse was essential for me*”, commented one respondent. “*Treatment so far from my community cost me a lot of money*”, wrote another. “*Financial help during and after treatment. Programs to help with financial loss of wages due to health reasons*” are needed, said another.

Access to timely, regular follow-up care and testing for recurrence was noted in 18.0% (571) of the comments regarding improvements. Close to a fifth of these comments highlighted the need for attentive healthcare providers and improved services, programs and facilities at cancer centers. In addition, there were 323 (10.2%) positive comments written, with the majority (66.9%, 216) submitted by older adults. These reflected respondents’ gratitude and appreciation for great, satisfying experiences and having their needs met with no concerns. “*I am completely satisfied with my treatment and follow up care*”, wrote one respondent. “*I am unaware of anything more than could have been done to help me*”, said another.

### 3.6. Comparison across Age Groups

Comparison of perspectives across age groups of rural-dwelling cancer survivors revealed similarity in both the types and frequency of comments from each group. Major challenges were most frequently identified as physical in all groups. The majority (55.9%) of the physical concerns were submitted by older adults who, given age and comorbidities, were likely to experience the most impactful physical issues; they also submitted 61.9% of the comments related to lifestyle adjustments. Adults (30 to 64 years of age) submitted 55.8% of the emotional concerns and 58.7% of the practical concerns. Adults in the age range where many would still be raising families and carrying significant financial responsibilities were understandably concerned about recurrence and issues transitioning back to work.

The most frequent positive experiences focused on the importance and value of helpful, attentive healthcare providers and guidance with self-care and support. Older adults submitted 57.1% of the total positive experiences, 61.8% provided advice to others/self-care comments that included the importance of maintaining a positive attitude, and 70.1% of the comments related to the positive impact of support from family and friends.

The top suggestions for improvement from all ages were related to the need for better information about side effect management, physical and mental concerns, and programs and services. Emotional and practical support was also highlighted. While responses from adults aged 30 to 64 represented 47.2% of the total, their suggestions comprised more than 50.0% of the information and communication (51.9%), practical support (61.7%), follow-up care (50.8%) and healthcare providers (56.0%) categories.

## 4. Discussion

This secondary analysis was undertaken to explore the perspectives of rural-dwelling Canadian cancer survivors regarding the major challenges they experienced following the completion of their cancer treatment, as well as what they thought were positive experiences and needs for improvements in survivorship care. The respondents included individuals from across Canada, thus under different jurisdictions for healthcare, and provided a sizeable sample of survivors living in rural areas. The study was the first cancer survivorship study of its kind in this country and provided insight into perspectives regarding the early survivorship period. Because rural residence has been identified as a barrier for patients during screening, diagnosis and treatment of cancer, this work was undertaken to garner increased understanding about its influence following the completion of cancer treatment for survivors in Canada. As an early exploration, a qualitative descriptive approach was deemed relevant. Additionally, the existing database provided an opportunity to engage in this secondary analysis.

Overall, it was anticipated that there would be considerably more comments written about living in a rural setting and having to confront issues unique to rural living. Specifically, we expected that travel and costs related to transportation and accommodation for follow-up treatment might emerge as a main challenge. However, this was not the case. The survey question about challenges requested respondents to name only one major challenge. Given that instruction, the priority for these survivors may no longer focus on travel. Over 70% of the respondents in this sample were more than one year after the completion of their treatment, and the need for frequent cancer clinic appointments, as was required during active treatment, was likely reduced. Follow-up appointments are likely to be scheduled on patterns of 3-month, 6-month or 12-month intervals. Additionally, some of the necessary follow-up tests (e.g., blood work, x-rays) can be acquired in the local/district hospitals, hence not necessitating the lengthy travel to regional or provincial clinics. There has been considerable effort in this country to move follow-up cancer care that could be handled in local communities by general practitioners or nurse practitioners to those locations [28,29]. Additionally, much of the medication, supplies and equipment possibly required during the early diagnostic and treatment interval may no longer be necessary, and easy access to them is not an issue.

The identification of physical issues as the major challenge emerged most frequently as the pressing issue for all age groups in this analysis. Physical change after cancer treatment is likely a priority for the survivors, given the interference limitations may have on activities of daily living, return to work and regaining a previous level of function regardless of where one lives. However, there may be added challenges in rural settings due to geographical distances, lack of convenient public transportation systems and fewer readily available neighbors to provide assistance. Further work is needed to explore this notion more deeply or contrast it with urban-dwelling survivors.

Limitations to one’s life and regaining expected functional performance can be a source of frustration and have an impact on quality of life. Physical limitations or reduced capacity to perform activities of daily living have been reported as issues by other cancer survivors [30,31,32,33]. The capacity to return to work for the AYA and adult groups and to maintain or regain independence for older adults have been cited as important goals for survivors in these age groups [34,35]. Throughout the respondents’ comments, the challenge of trying to return to one’s previous way of life was an underlying expectation. This notion could be observed in the comments regarding reduced physical capacity to engage in activities, the need for lifestyle adjustment and frustrations at slow recovery, as well as the need for improvements in services to assist with these types of challenges. Access to services by physiotherapists, exercise trainers and other rehabilitation professionals were cited together with the desire to have these services available close to home.

It is also important to note that the proportion of older adults in this sample likely influenced the types of issues that were identified most frequently. Many older adults are dealing with comorbidities in addition to the impact of their cancer. In this sample, 62.1% (2887) overall reported comorbidities, including those that would likely result in pain and limited mobility, such as cardiovascular or heart conditions, arthritis, osteoarthritis, and diabetes. In reporting symptoms, it may be difficult for the survivors to differentiate between the symptoms resulting from the cancer and those related to the comorbidities [36].

Reflecting on what constituted positive experiences, this rural sample identified the value of family and friend support. Many comments embodied how grateful and thankful survivors were for their spouses and partners as well as other family members. This observation has also been cited by other survivors [4,15,19] in general but has not been contrasted with the experiences of urban-dwelling cancer survivors. However, it could be particularly relevant in a rural situation to offer practical assistance in the home or on a farm. Reliance on family or nearby neighbors is cited as a strong aspect of rural community life [23,24], but whether or not there are unmet needs concerning assistance in rural settings would require future study.

The respondents also emphasized the importance of knowledgeable and compassionate healthcare professionals as an important aspect of what made their experiences positive. Their perspectives were similar to those reported by many cancer patients [37,38,39,40]. Comments from these survivors described how individual healthcare professionals interacted with them in ways that made the survivors feel heard and that their needs were seen as important. Previous investigations regarding how patients “see” quality of care also emphasized these elements [37,38,39]. In particular, the experiences of being listened to, having others understand the perspectives of the patient or survivor, and being treated with dignity and compassion are repeated in studies focused on patient perspectives on quality care [38,40]. The importance of communication and support has also been well articulated by other survivors [41,42]. Many of the suggestions for improvement at cancer clinics reflected the need for consideration of ways to achieve better communication, compassionate interactions, and clarity around what was expected or anticipated in follow-up care. In essence, these improvements reiterate what other cancer patients have cited [18,43,44], emphasizing the need for the cancer system to respond in more meaningful ways regarding person-centered care [45,46].

Many of the suggestions for improvement during the early survivorship period by these survivors spoke to the need for information and support for the survivor and the family. Once again, these perspectives reflect what other cancer patients have reported [38,39]. Both suggestions could reflect an attitude of, or desire for, self-sufficiency and being able to move on with life. Armed with information about what to expect (e.g., treatment and medication side effects, self-care tips, expectations for full recovery, etc.) and available services for support (practical, financial, emotional) following cancer treatment, individuals would be in a better position to engage in self-care. From the rural-dwelling respondents, these programs would be most helpful if they were close to their home setting or, in some cases, online for easy access.

## 5. Limitations

Several limitations exist with this analysis. Confidentiality issues limited information about survivors that could be shared from the registry, leaving insufficient detail to allow weighting of survey results to have them representative of all Canadian survivors of cancer. The intention of sampling was to target five disease sites and survivors one to three years post-treatment, yet self-reported survey data revealed that just under 10% of survivors indicated they had a cancer type outside the five targeted originally. A total of 24.7% of the rural respondents were more than three years post-treatment, and 16.1% indicated they had not received treatment or follow-up care. Over 3.2% of respondents did not disclose their residential location in the original Transitions Study, and there was no way to assess whether the missing data were random over this variable. The Transition Study sample does not reflect the distribution of individuals living in rural locations across Canada, as reported by Statistics Canada (17.8% of the total Canadian population) [21]. However, our definitions of rural dwelling and that of Statistics Canada differ. Hence, results from this secondary analysis cannot be generalized to the Canadian population living in rural settings.

This secondary analysis was an exploration using a publicly available dataset first created in 2026, thus imposing limitations on the data available for incorporation into this work and the need to acknowledge there have been changes in healthcare in this country following the COVID-19 pandemic. Future analysis would benefit from including educational and occupational data and incorporating other social determinants of health. Finally, there is a limitation in the use of written data, as there was no opportunity to ask the respondents to elaborate on their perspectives.

## 6. Implications

Based on the results of this analysis, the need for healthcare professionals to be aware of the potential challenges for survivors, especially those living rurally, is important. Clinicians should consider assessments of risk for experiencing difficulties and intentional conversations about the possible impact of issues during survivorship. There is also a need to consider offering programs or referrals to enhance resilience and positive coping. Cancer centers may also need to consider approaches to the provision of information regarding available support programs as part of standard care practices.

Additionally, the results of this analysis emphasize the need for cancer programs and policy makers to explore support programming and its availability in local communities. New collaborations may be warranted with existing services so that cancer survivors have increased access to assistance and support close to home after their frequent appointments at the cancer clinics are reduced.

## 7. Conclusions

Rural-dwelling Canadian survivors identified physical changes following cancer treatment most frequently as the major challenge. Their positive experiences and suggestions for improvement were focused primarily on relationships with families and healthcare providers who were knowledgeable and compassionate. The need for information to anticipate possible challenges that could occur during survivorship and what could be performed to address them was emphasized as important. Ideally, survivors wanted more programs available locally to support practical and emotional needs following the completion of cancer treatment.

## Figures and Tables

**Table 1:** Respondent profile (n = 4646).

Variable	Number	Percentage
Sex		
Male	2327	50.10%
Female	2296	49.40%
No answer	23	0.50%
Age		
18–29	69	1.50%
30–64	1638	35.30%
65 and older	2926	63.00%
No answer	13	0.30%
Marital Status		
Single	232	5.00%
Married/partnered	3581	77.10%
Separated/divorced/widowed	794	17.10%
Prefer not to answer	39	0.80%
Education		
High School or less	3059	65.40%
Post-secondary degree (college/university)	1296	27.90%
University graduate degree	222	4.80%
Missing	89	1.90%
Disease site *		
Breast	1305	28.10%
Prostate	1202	25.90%
Colorectal	970	20.90%
Melanoma	508	10.90%
Hematological	345	7.40%
Other	187	4.00%
Missing	250	5.40%
Metastases		
No metastases	3522	75.80%
Living with metastases	440	9.50%
Unsure	414	8.90%
Missing	270	5.80%
Time since treatment		
<1 year	472	10.20%
1 year to <3 years	2107	45.40%
3 years or more	1148	24.70%
Did not receive treatment	747	16.10%
Missing	172	3.70%
General physical health (one item)		
Very poor/poor	183	3.90%
Fair	984	21.20%
Good/very good	3458	74.40%
Missing	21	0.50%
General emotional health (one item)		
Very poor/poor	167	3.60%
Fair	777	16.70%
Good/very good	3464	74.60%
Missing	238	5.10%
Overall quality of life (one item)		
Very poor/poor	83	1.80%
Fair	721	15.50%
Good/very good	3823	82.30%
Missing	19	0.40%
Comorbidities		
Yes	2887	62.10%
No	1566	33.70%
Missing	193	4.20%
Comorbidities (4 most common)		
Cardiovascular or heart condition; hypertension or high blood pressure	1417	30.50%
Arthritis, osteoarthritis, or other rheumatic disease	1406	30.20%
Diabetes	555	11.90%
Mental health issues	440	9.50%

* Percentages add to greater than 100 because respondents could select multiple sites.

**Table 2:** Major challenges.

Major Challenges n = 5448			Number of Comments by Age Groups			Topic Total	Percentage of Topic
Categories	Number	Sub-Topics	AYA 18–29 n = 120	Adults 30–64 n = 2425	Older Adults 65+ n = 2903		
PHYSICAL	3493 (64.1%)	Capacity (fatigue/mobility)	21	527	611	1159	33.2%
		Pain/numbness/swelling	11	250	207	468	13.4%
		Other side effects *	11	147	262	420	12.0%
		Therapy effects **	3	153	193	349	10.0%
		Urological effects	0	76	195	271	7.8%
		Bowel problems	0	65	186	251	7.2%
		Sexual/fertility concerns	4	79	119	202	5.8%
		Post-surgical complications	4	67	94	165	4.7%
		Body image	5	51	64	120	3.4%
		Cognitive effects	8	58	22	88	2.5%
EMOTIONAL	713 (13.1%)	Emotional issues, coping ***	11	153	120	284	39.8%
		Fears (recurrence/death)	4	143	107	254	35.6%
		Depression/anxiety/stress	7	102	66	175	24.5%
SERVICE DELIVERY	376 (6.9%)	Information/communication	2	66	67	135	35.9%
		Follow-up care	0	51	54	102	27.9%
		Healthcare providers	1	34	39	74	19.7%
		Hospital/clinic services	0	30	32	62	16.5%
LIFESTYLE ADJUSTMENTS	344 (6.4%)	Managing change/normality	5	93	159	257	74.7%
		Difficulty eating	2	16	35	53	15.4%
		Difficulty sleeping	1	14	19	34	9.9%
PRACTICAL	269 (4.9%)	Return to work/school	13	95	16	124	46.1%
		Chores/transportation help	0	23	52	75	27.9%
		Financial concerns	2	40	28	70	26.0%
RELATIONSHIPS, SUPPORT	102 (1.9%)	Family challenges/concerns	1	29	23	53	52.0%
		Lack of emotional support	0	20	14	34	33.3%
		Challenges with friends	1	6	8	15	14.7%
NO CHALLENGES	76 (1.4%)	No challenges reported	2	19	55	76	100.0%
OTHER	45 (0.8%)	No or still in treatment	0	7	38	45	100.0%
POSITIVE	30 (0.6%)	Positive comments	0	13	17	30	100.0%

* Other side effects of therapy; examples: long recovery, comorbidities, infection, weight gain/loss. ** Therapy effects; medication, chemotherapy, radiation effects. *** Emotional issues/coping; examples: anger, low self-esteem/motivation, insecurity, vulnerabilities.

**Table 3:** Positive experiences.

Positive Experiences n = 3561			Number of Comments by Age Groups			Topic Total	Percentage of Topic
Categories	Number	Sub-Topics	AYA 18–29 n = 64	Adults 30–64 n = 1.461	Older Adults 65+ n = 2036		
HEALTHCARE PROVIDERS	809 (22.7%)	Excellent/knowledgeable HCPs	9	191	205	405	50.1%
		Attentive, compassionate, caring HCPs	12	153	152	317	39.2%
		Good access to HCPs	3	37	35	75	9.3%
		Support from HCPs	0	7	5	12	1.5%
SELF-CARE/ADVICE TO OTHERS	759 (21.3%)	Stay positive, confident	1	82	175	258	34.0%
		Ask for help/trust HCPs	1	118	132	251	33.1%
		Other (stay calm/healthy)	5	52	105	162	21.3%
		Have faith/live each day	3	28	57	88	11.6%
SUPPORT	736 (20.7%)	Family and friends support	2	104	260	366	49.7%
		Peer and group support	3	116	130	249	33.8%
		Help from others/HCPs	1	45	34	80	10.9%
		Faith/spiritual support	0	4	27	31	4.2%
		Practical support	1	3	6	10	1.4%
FOLLOW-UP CARE	312 (8.8%)	Care by doctors, oncologists, surgeons	2	76	90	168	53.8%
		Routine tests/home visits	1	25	49	75	24.1%
		Regular/timely follow-up	1	29	39	69	22.1%
INFORMATION AND COMMUNICATION	256 (7.6%)	Good communication/trust with HCPs	1	76	108	185	72.3%
		Information on side effects, healthy living	0	16	27	43	16.8%
		Education forums, workshops, programs	0	15	13	28	10.9%
CANCER CENTRES	166 (4.7%)	Good clinics/schedules	0	40	33	73	44.0%
		Quick/timely diagnosis, treatment, test results	1	28	16	45	27.1%
		Access to services/ treatment close to home	0	16	16	32	19.3%
		Accommodation for out-of-town patients (lodges)	0	4	12	16	9.6%
COMPLIMENTARY/SUPPORT THERAPIES	130 (3.7%)	Guidance/help with diet, exercise, sleep	0	12	40	52	40.0%
		Alternative therapies	0	38	14	52	40.0%
		Restorative, rehabilitation therapies	2	10	14	26	20.0%
NO POSITIVE EXPERIENCES	168 (4.4%)	Nothing positive to report	7	56	105	168	100.0%
POSITIVE	142 (4.0%)	Successful treatment, good experience	4	57	70	131	92.3%
		Recovery, returning to normal	0	5	6	11	7.7%
OTHER	83 (2.3%)	No follow-up care required	4	18	61	83	100.0%

**Table 4:** Suggestions for improvements.

Suggested Improvements n = 3181			Number of Comments by Age Groups			Topic Total	Percentage of Topic
Categories	Number	Sub-Topics	AYA 18–29 N = 68	Adults 30–64 N = 1503	Older Adults 65+ N = 1610		
INFORMATION AND COMMUNICATIONS	798 (25.1%)	Information				683	85.6%
		Self-care, recurrence, care plans, condition	5	137	91	233	
		Side effects/post-treatment issues	2	103	121	226	
		Programs/services/support groups	2	95	61	158	
		Other (better information, general, unspecified)	1	30	35	66	
		Communication				115	14.4%
		With/among HCPs	2	49	51	102	
		In my language	0	0	13	13	
SUPPORT/SELF-CARE	574 (18.0%)	Support—General				216	37.6%
		Services/groups (plus peer)	4	72	41	117	
		Family/friends support	3	24	35	62	
		Community, phone support	1	13	23	37	
		Support—Practical				206	35.9%
		Financial aid	7	73	48	128	
		Return to work issues	0	41	3	44	
		Help with chores/travel	1	13	20	34	
		Support—Emotional				52	19.5%
		Personal/one on one	1	14	14	29	
		Help with issues, coping	2	12	9	23	
		Self-care				40	7.0%
		Be your own advocate	1	13	15	29	
		Other (faith, positivity)	2	1	8	11	
FOLLOW-UP CARE	571 (18.0%)	Access to/care by HCPs	2	73	90	165	28.9%
		Timely/regular/care	2	77	80	159	27.8%
		Post-treatment therapies	12	85	52	149	26.1%
		Symptom management	2	55	41	98	17.2%
HEALTHCARE PROVIDERS	318 (10.0%)	Attentive, compassionate HCPs	5	64	44	113	35.5%
		Good/knowledgeable HCPs	1	40	48	89	28.0%
		Access to/more time with HCPs	1	50	26	77	24.2%
		Coordination among HCPs	0	24	15	39	12.3%
CLINICS/HOSPITAL SERVICES	228 (7.2%)	Improved services/facilities/closer to home	0	59	81	120	61.4%
		Shorter wait times for results/appointments	1	23	37	61	26.8%
		Better appointment schedules, patient choice	0	14	13	27	11.8%
POSITIVE COMMENTS	323 (10.2%)	Great care, satisfying experience	4	89	180	273	84.5%
		No concerns, needs met	1	13	36	50	15.5%
NO SUGGESTIONS	314 (9.9%)	No suggestions	3	88	223	314	100.0%
OTHER	52 (1.6%)	No follow-up care required	0	13	39	52	100.0%
NEGATIVE COMMENTS	3 (0.1%)	Negative comments	0	2	1	3	100.0%

## Data Availability

Canadian Partnership Against Cancer has full control of primary unidentifiable record-level data. The dataset is publicly available (https://www.systemperformance.ca/transition-study/). Accessed 1 January 2020.
